# Onset of normal cycles in postpartum anovulatory dairy cattle treated with kisspeptin

**DOI:** 10.1530/RAF-21-0046

**Published:** 2021-12-20

**Authors:** Chris R Burke, John R Roche, Robert P Millar, Iain J Clarke

**Affiliations:** 1 DairyNZ Ltd., Hamilton, New Zealand; 2 Centre for Neuroendocrinology, Department of Immunology, University of Pretoria, Gezina, Pretoria, South Africa; 3 University of Monash, Clayton, Victoria, Australia

**Keywords:** kisspeptin agonist, ovulation, postpartum dairy cow

## Abstract

The efficacy of a long-acting synthetic derivative of kisspeptin (Kp) to initiate normal oestrous cycles was tested in 24 mixed-aged, Holstein-Friesian cows that were 18–25 days postpartum on the day of treatment (D0). Groups of eight cows received saline (Sal) vehicle by intramuscular injection at 8:00 and 16:00 h (Sal-Sal), Kp at 8:00 h and vehicle at 16:00 h (Kp-Sal) or Kp on both occasions (Kp-Kp). The Kp dose was 15 nmol per 60 kg body weight. The ovaries of the cows were examined daily by ultrasonography between D4 and D14. Blood samples were collected from a tail vessel at 0, 2, 4, 8, 10 and 12 h relative to the time of the first injection for luteinizing hormone (LH) and follicle-stimulating hormone assay. Additional samples were collected daily from D4 until D14 and D19, 22, 26 and 29 for progesterone assay. LH surge-like responses were observed in cows treated with Kp at 8:00 h. Ovulation was consistently induced by Kp within 48 h when a dominant ovarian follicle of at least 10 mm in diameter was observed (8/14) but in no cases (6/14) during a new wave of ovarian follicular development comprising follicles <10 mm in diameter. The subsequent ovulatory cycle was of normal length in most cases as compared with short 8– to 12-day cycles observed in spontaneously ovulating cows. We conclude that Kp treatment can induce ovulation in postpartum dairy cows, with ensuing oestrous cycles of normal length, if administered when a mature dominant follicle is present in the ovaries.

**Lay summary:**

Cow fertility is important for efficient, profitable dairy farming. Cows that take too long after calving to become fertile are problematic. We tested a synthetically made, long-acting hormone called kisspeptin (Kp) to advance the time that cows become fertile after calving. Twenty-four dairy cows that had been calved for 3–4 weeks were used. One group of eight cows received an injection of Kp at the morning milking, another eight cows received Kp at both the morning and afternoon milking, while the last group of eight cows served as untreated controls. Kp treatment caused a desirable hormone response from the cows’ brain. Normal oestrous cycles resulted, but only when a mature follicle was present in the ovary. Further study is required to analyse whether the use of a long-acting Kp drug could be used as an effective treatment for stimulating dairy cows to become more fertile after calving.

## Introduction

Prolonged postpartum anovulation is a major cause of infertility in dairy cattle, particularly for seasonal production systems reliant on maintaining a 365-day calving interval (Fielden *et al.* 1973). While there are efficacious treatments available to treat non-cyclic cows (Rhodes *et al.* 2003), these involve multiple hormones and manipulations and tend, therefore, to be relatively expensive. An example is the intravaginal placement of a progesterone-releasing device for 7 days along with injections of a gonadotrophin-releasing hormone (GnRH) and prostaglandinF_2ά_ (McDougall *et al.* 2010). A simpler, lower-cost option for increasing the proportion of the herd displaying normal ovulatory cycles before the onset of a seasonal breeding period may overcome these disadvantages and offers producers a more desirable treatment proposition.

A previous report (McDougall *et al.* 1995*b*) indicated that a luteinizing hormone (LH) surge and ovulation is inducible using a single intramuscular injection of GnRH at 3 weeks postpartum, provided a dominant follicle >10 mm in diameter was present in the ovaries at the time of treatment. This study did not test the responses to GnRH when dominant follicles had recently emerged and were <10 mm in diameter. These functionally dominant, smaller follicles are unlikely to have acquired the capacity to ovulate following an LH surge (Ginther *et al.* 2001) and will be present among cows within a population being treated on any given day (McDougall *et al.* 1995*a*). Furthermore, as reported by McDougall *et al.* (1995*b*), most GnRH-treated cows did not sustain ovulatory cycles beyond a single, short-luteal phase of <10 days and questioned whether the hypothalamus of dairy cattle in early postpartum was capable of supporting gonadotrophin release to sustain a continuation of a cyclic state.

Kisspeptin (Kp) is a peptide produced in the brain, in the hypothalamus and in some peripheral organs, notably the gonads, uterus and placenta (Babwah 2015, Uenoyama *et al.* 2016). Kp neurons of the arcuate nucleus of the hypothalamus project into the external zone of the median eminence to come into close proximity to GnRH neurosecretory terminals outside the blood–brain barrier (Smith *et al.* 2011). Kp directly stimulates the secretion of GnRH (Smith *et al.* 2011), and endogenous pulsatile secretion of GnRH is driven by Kp neurons of the arcuate nucleus, which also initiates the preovulatory GnRH surge in ewes (Smith *et al.* 2009). GnRH secretion is stimulated by systemic administration of Kp (Caraty *et al.* 2013) and by direct injection into the median eminence (Ezzat *et al.* 2015). The stimulatory effect of Kp on the LH secretion has been demonstrated in several species, including cattle (Kadokawa *et al.* 2008, Ezzat Ahmed *et al.* 2009). Whereas the Kp neurons of the arcuate nucleus come into close association with GnRH neuronal terminals in the median eminence (Smith *et al.* 2011, Clarke 2015), a second population of Kp neurons is found in the preoptic area of the ruminant (Franceschini *et al.* 2006, Hassaneen *et al.* 2016). These rostrally located Kp neurons, along with GnRH neurons in the same region, display Fos labelling after the start of the GnRH surge (Hoffman *et al.* 2011), indicating their role in facilitating the preovulatory surge of GnRH (Clarke 2017).

Longer-acting, more potent derivatives of the native Kp have been designed for therapeutic applications (Curtis *et al.* 2010, Asami *et al.* 2013, Decourt *et al.* 2016). Of interest to us was the Kp agonist described by Maclean *et al.* (2014) and whether a depot injection approach, rather than continuous infusion, could advance the onset of normal-length oestrous cycles in postpartum dairy cows where maturity state of the ovarian dominant follicle is variable.

## Materials and methods

Prior approval was gained for animal experimentation by the Ruakura Animal Ethics Committee, and the use of a Kp agonist was approved by the Agricultural Compounds and Veterinary Medicines Group, Ministry for Primary Industries, New Zealand. The agonist we used is identical to TAK448 (Asami *et al.* 2013, Maclean *et al.* 2014). It was produced by Anatech (Cape Town, South Africa) using conventional solid-phase peptide synthesis followed by HPLC purification. A single peak on HPLC confirmed purity at >90%. Mass spectrometry revealed a mass ion of 1225 g/mol, confirming structure and composition as the targeted Kp agonist; Ac – Y(D)-Hyp-N TF-X LR(N-Me)W-NH_2_. This agonist was validated to elevate LH for 5 h in ewes during the luteal phase of the oestrous cycle (IJ Clarke, unpublished observation).

Thirty-five lactating dairy cows aged 2–4 years that were 18–25 days postpartum were initially enrolled to the study conducted at the DairyNZ Scott Research Farm, Hamilton, New Zealand. The development of ovarian follicles was monitored daily at 8:00 h by transrectal ultrasonography (5–15 MHz probe – SonoScape S6V, Euromed Medical Systems, Auckland, New Zealand) after the morning milking for 3 days. Individual follicles ≥3 mm in diameter and any corpora lutea on the ovaries were identified. Diameter of these structures was assessed with the aid of in-built screen callipers and transcribed to recording sheets. Information from these recording sheets was then used to construct graphical maps for each ovary of every cow for each observation day. These maps were then used to determine the stages of follicular wave development (e.g. new wave emergence, establishment of dominance and sudden disappearance of dominant follicles).

Twenty-four cows, which displayed no corpora lutea on the ovaries prior to treatment administration, were selected for final enrollment. As a population, they were 25.4 days (s.d. 2.6) postpartum, producing 20.1 kg (s.d. 3.2) milk per day and with a body weight of 426 kg (s.d. 47) and a body condition score (BCS) of 4.3 units (s.d. 0.3) on a 1–10 scale (Roche *et al.* 2004). Allocation to treatment groups was made by first blocking the cows by diameter of the dominant follicle on the day before treatment administration. The preceding 3 days of daily ovarian ultrasonography enabled the identification of the dominant follicle and its diameter. In the case of new follicular wave emergence, the largest follicle was assumed as the dominant follicle. There were three blocks used; dominant follicle <12 mm, 12–15 mm and 16–18 mm. Cows from each block were then randomly assigned to one of three treatment groups, and one-way ANOVA was used to assess whether the groups differed in age, days postpartum, BCS and live weight. The respective *P*-values were 1.0, 0.69, 0.33 and 0.59, and no further adjustments were made to the treatment group allocation. Descriptive statistics for treatment groups are presented in Table 1. Treatments were administered on the fourth day (designated D0) by intramuscular injection at 8:00 and 1600 h as follows: Sal-Sal – saline ‘vehicle’ on both occasions (control group); Kp-Sal – Kp in the morning and saline in the afternoon and Kp-Kp – Kp in both morning and afternoon. Dosage of Kp was 15 nmol per 60 kg body weight and based on that we found the desired LH response to be provided in the sheep (IJ Clarke, unpublished observation). A 420-kg cow, for example, received 105 nmol Kp per dose in the current study. Ovarian ultrasonography was then performed daily at 8:00 h for 14 days, and cows were monitored daily for signs of behavioural oestrus with the aid of tail paint (Macmillan *et al.* 1988).

**Table 1:** Descriptive average (and s.d.) values on day of treatment administration (D0) for days since calving, milk yield, body weight, body condition score (BCS) and diameter of the dominant follicle. Treatments were administered by intramuscular injection at 8:00 and 16:00 h, as follows: Sal-Sal – saline ‘vehicle’ on both occasions (control group); Kp-Sal – Kp agonist in the morning and saline in the afternoon; and Kp-Kp – Kp agonist in both morning and afternoon. Dosage of Kp agonist was 15 nmol per 60 kg body weight.

	Sal-Sal	Kp-Sal	Kp-Kp
Cows allocated (*n*)	8	8	8
Days since calving	24.8 (2.6)	25.8 (2.5)	25.8 (2.8)
Milk yield (kg/day)	21.1 (3.3)	19.8 (2.7)	18.9 (4.2)
Body weight (kg)	412 (44)	437 (44)	429 (56)
BCS (1–10 scale)	4.1 (0.2)	4.4 (0.4)	4.3 (0.4)
Diameter of dominant follicle (mm)	12.0 (3.7)	9.4 (4.5)	12.3 (5.0)

Blood samples were collected by coccygeal venepuncture into Li heparin tubes (Vacutainers, BD, Auckland, New Zealand) held in iced water for LH and follicle-stimulating hormone (FSH) assay at 0, 2, 4, 8, 10, 12 and 24 h relative to the time of the first injection. Additional samples were collected daily from D4 until D14 and on D19, 22, 26 and 29. This sampling regimen extended 14 days beyond the daily ultrasonography period to assess the oestrous cycle lengths. The samples were centrifuged (1500 ***g*** for 12 min at 4°C) and harvested plasma was stored at −20°C until assayed for progesterone, LH and FSH as appropriate.

A commercial double antibody RIA kit was used to determine plasma progesterone concentrations in accordance with the manufacturer’s instructions (ImmuChem Double Antibody Progesterone RIA, ICN Biomedicals, Costa Mesa, CA). The standard curve ranged from 0.2 to 50 ng/mL. Samples were assayed in duplicate. The assay had a sensitivity of 0.2 ng/mL and an intra-assay coefficient of variation (CV) of <10% between 0.3 and 16.4 ng/mL. The inter-assay CV (three assays) for pooled bovine plasma samples with mean concentrations of 0.75 and 3.8 ng/mL were 14.8 and 9.2%, respectively. The minimal detectable concentration was 0.2 ng/mL. Plasma LH concentrations were measured in a single RIA using NIH, AFP-11118 as a standard. The standard curve ranged from 0.5 to 50 ng/mL. Samples were assayed in duplicate. The assay had a sensitivity of 0.1 ng/mL and an intra-assay CV of <10% between 0.6 and 18.5 ng/mL. Samples with values below the level of sensitivity were assigned 0.05 ng/mL for data analysis. Plasma FSH concentrations were measured in a single RIA using NIH, AFP-9294C as a standard. The standard curve ranged from 0.5 to 50 ng/mL. Samples were assayed in duplicate. The assay had a sensitivity of 0.1 ng/mL and an intra-assay CV of <10% between 1.2 and 11.4 ng/mL. Samples with values below the level of sensitivity were assigned 0.05 ng/mL for data analysis.

Three cows (one per treatment group) were retrospectively identified as having ovulated immediately prior to D3 and were omitted from further analyses. Incidence of ovulation among the remaining cows (*n*  = 7 per group) was determined using the daily ovarian ultrasonography data and was defined as the disappearance of a dominant follicle coupled with subsequent development of a corpus luteum and elevated concentrations of progesterone. Day of ovulation was when a dominant follicle was observed to have disappeared.

The trial was designed to investigate whether the time course of LH and FSH appearance in blood is affected by the treatment. The statistical analyses performed were in line with the main hypothesis and the design of the trial but were not formally outlined in a statistical analysis plan. The LH and FSH data were log_10_ transformed for analysis to achieve homogeneity of variance after visual assessment of standardized residual plots. Responses were analysed using a mixed model approach to repeated measures ANOVA (Proc Mixed, SAS 9.4). The model included treatment, time and their interaction as fixed effects and cow as random effect. A spatial power covariance (SP(POW)) structure pattern model was used. This ANOVA was followed by pairwise comparisons between groups within time using Tukey’s multiple comparison procedure. A one-way ANOVA was used to test whether diameter of the dominant follicle at D0 differed among the groups. Significance was declared if *P* ≤ 0.05 and a trend was declared if *P* > 0.05 ≤ 0.1. All other data are considered descriptive and have not been subjected to statistical testing due to low sample sizes.

## Results

**Figure 1:**
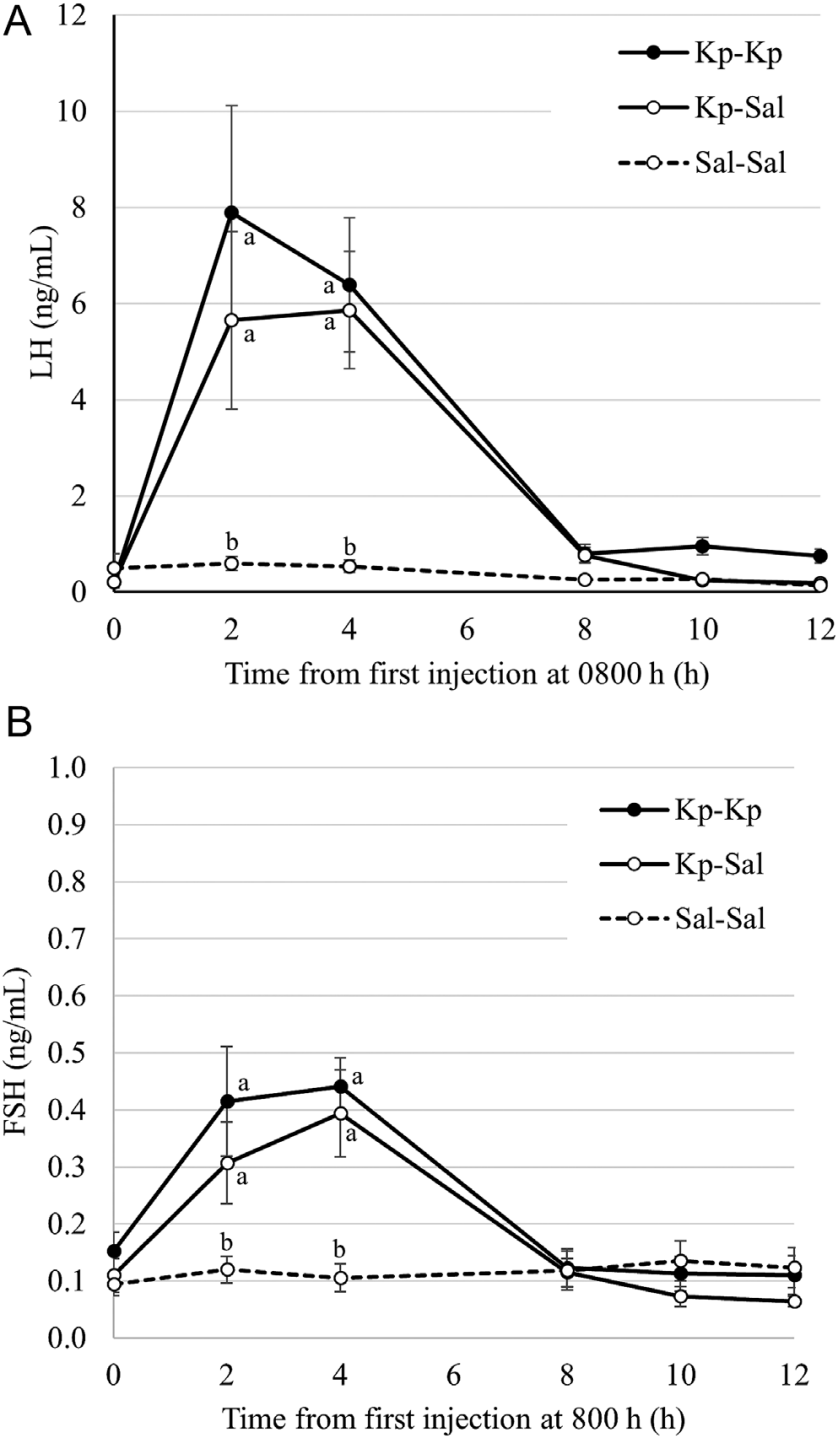
Mean plasma concentrations of LH (A) and FSH (B) in cows receiving intramuscular injection at 8:00 and 16:00 h of saline vehicle (Sal-Sal; *n*  = 7), Kp at 8:00 h and saline at 16:00 h (Kp-Sal; *n*  = 7) or Kp at 8:00 h and 16:00 h (Kp-Kp; *n*  = 7). Cows that ovulated prior to treatment administration are excluded. Error bars are s.e.m. The effects of treatment group, time and the group × time interaction on LH and FSH responses were all significant (*P* < 0.01). Different letters within timepoints denote difference at *P*< 0.01.

A treatment group × time interaction (*P* < 0.01) was detected for LH response. Administration of Kp at 8:00 h increased plasma concentrations of LH (Fig. 1A), and this was significant (*P* < 0.01) at the 2- and 4-h time points when comparing Sal-Sal with either Kp-Sal or Kp-Kp. There were no differences in LH between the two Kp groups at any time point and no differences among any of the three groups beyond 4 h.

A treatment group × time interaction (*P* < 0.01) was detected for FSH response (Fig. 1B), which resembled that of the LH response. Concentration of FSH was greater (*P*< 0.01) at 2 and 4 h in the Kp-Sal and Kp-Kp groups, respectively compared with the Sal-Sal controls. There were no differences in FSH between the two Kp groups at any time point and no differences among any of the three groups beyond 4 h.

**Figure 2:**
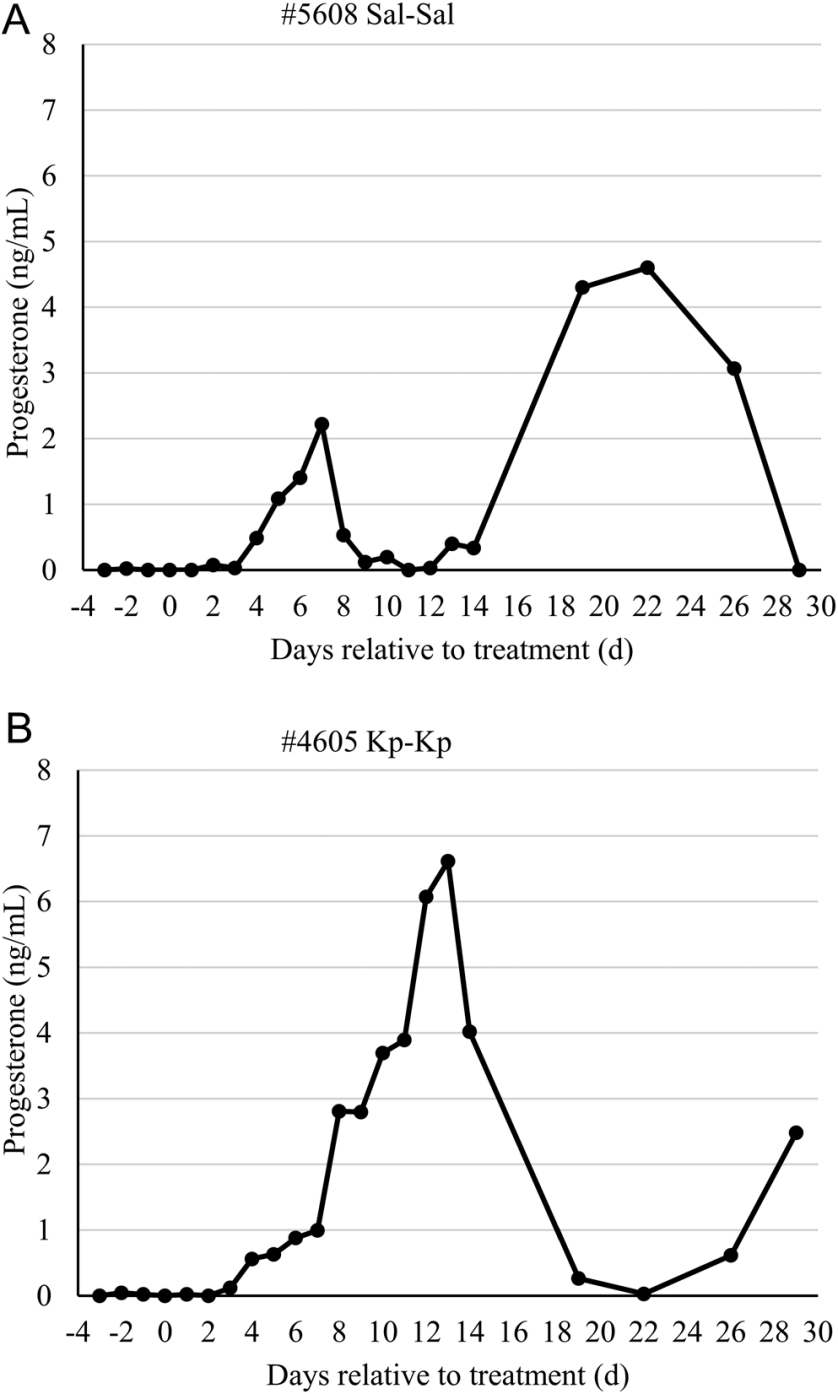
Representative plasma concentrations of progesterone in a Sal-Sal control cow (#5608) that spontaneously ovulated (A) and a Kp-treated cow (#4605) induced to ovulate (B).

**Figure 3:**
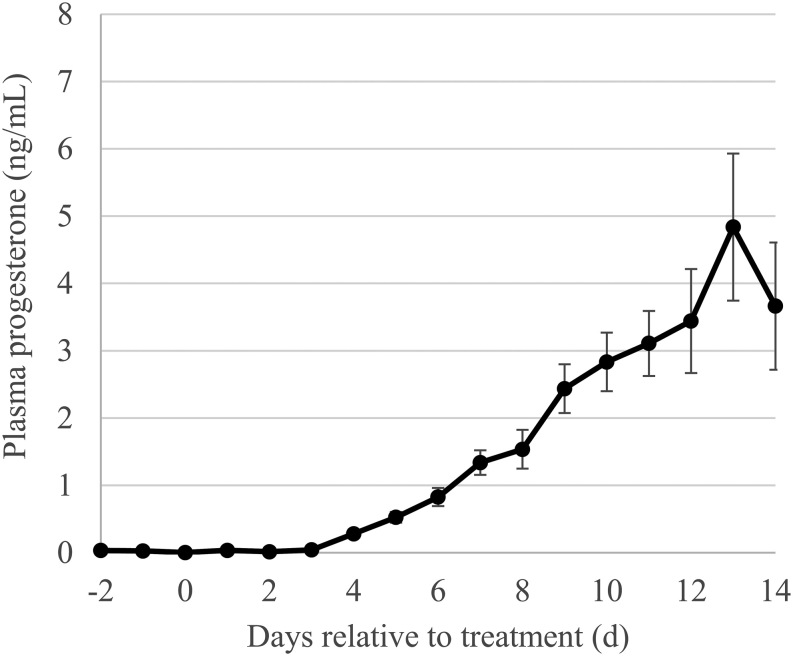
Average (± s.e.m.) daily concentrations of plasma progesterone at D14 among six of eight cows that ovulated in response to Kp treatment and had normal-length oestrous cycles.

The diameter of the dominant follicle at D0 was not different (*P* = 0.43) among the treatment groups (Table 1), with an overall average of 11.2 mm (s.e.m. 1.0) and a range from 4 to 18 mm. A description of responses to the treatment is presented in Table 2. Within the Sal-Sal (control) group, three cows ovulated spontaneously (on D2, 9 and 11, respectively) and four remained anovulatory until D29. All three cows that ovulated had short oestrous cycles (8–12 days) followed with cycles of normal length (Fig. 2A). Among the Kp-treated groups (i.e. Kp-Sal and Kp-Kp), 8 of 14 cows ovulated on D1 (within 48 h of treatment), 1 cow ovulated on D9 and 5 cows remained anovulatory until D29. None of the Kp-induced ovulations, or first postpartum spontaneous ovulations, were accompanied with detected behavioural oestrus. In six of the eight Kp-treated cows induced to ovulate within 48 h, the subsequent oestrous cycle was more ‘normal’ in length, as an example in Fig. 2B depicts. The average daily concentrations of progesterone until D14, for these six cows, are presented in Fig. 3. The remaining two cows that responded to Kp had oestrous cycles resembling the short 8–12-day length, as for the example depicted in Fig. 2A.

**Table 2:** Descriptive responses for ovulation and oestrus, oestrous cycle duration and anovulatory state following treatment administration (D0). Treatments were administered by intramuscular injection at 8:00 and 16:00 h as follows: Sal-Sal – saline ‘vehicle’ on both occasions (control group); Kp-Sal – Kp agonist in the morning and saline in the afternoon; and Kp-Kp – Kp agonist in both morning and afternoon. Dosage of Kp agonist was 15 nmol per 60 kg body weight.

	Sal-Sal	Kp-Sal	Kp-Kp
Cow number (*n*)^1^	7	7	7
Dominant follicle <10 mm diameter on D0	2	4	3
Dominant follicle 10–18 mm diameter on D0	5	3	5
Ovulated 24–48 h after D0	1	3	5
Ovulation accompanied with oestrus	0	0	0
Oestrous cycle duration >12 days	0	2	4
Remained anovulatory at D29	4	3	2

^1^Excludes cows (one per treatment group) retrospectively identified as having ovulated before treatment administration based on observed development of corpora lutea.

**Figure 4:**
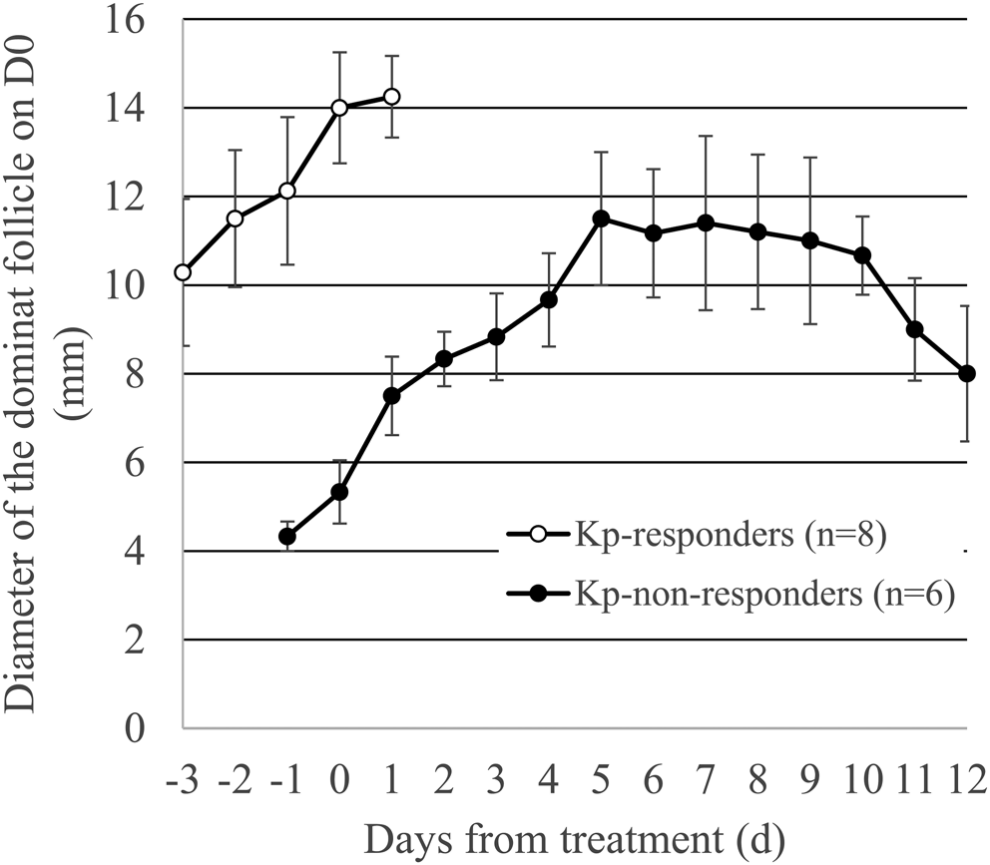
Average daily diameter of the dominant follicle (± s.e.m.) among Kp-treated cows that ovulated in response to Kp (open circles; *n*  = 8) compared with those that failed to ovulate the dominant follicle present at the time of Kp administration (closed circles; *n*  = 6).

Maturity of the dominant follicle at D0 determined the ovulatory outcome to Kp treatment (Table 2). Ovulation occurred in all cases where a dominant follicle of at least 10 mm in diameter was present in the ovaries (8 of 14 cows), whereas cows remained anovulatory until D29, when Kp was administered coincident with a new wave of follicle development lacking a dominant follicle of at least 10 mm in diameter (6 of 14 cows). The effect of dominant follicle maturity at D0 on ovulatory responses is depicted in Fig. 4. Among the Kp-treated cows, average daily diameter for those eight cows that ovulated was greater compared with those six cows with newly emerged dominant follicles at D0 that failed to ovulate.

## Discussion

While there were a limited number of observations to test the hypothesis that Kp would be an effective agent for induction of ovulation and normal oestrous cycles in the early postpartum period, results of the current study provide a useful indication of what responses are expected from treating anoestrous dairy cows with a long-acting derivative of Kp.

Previous reports on responses of Kp administration in cattle (Leonardi *et al.* 2018, 2020, Macedo *et al.* 2019) described work with native forms of Kp that are less potent and shorter acting compared with the Kp agonist derivative used in the current study. The dosage we tested (equivalent to 0.25 nmol/kg body weight) was substantially lower than that used by Leonardi *et al.* (2020; 45 mg per 476 kg live weight equivalent to 80 nmol/kg body weight) and Macedo *et al.* (2019; 2.5–10 μg Kp per Kg body weight equivalent to 5000–20,000 nmol/kg live weight). Administration of the first Kp agonist injection in the current study elevated LH in circulation to approximately 6 ng/mL for at least 4 h, followed by a return to basal concentrations by 8 h. This contrasts with the response to native forms of Kp, where LH concentrations remain elevated for less than 3 h (Ezzat Ahmed *et al.* 2009, Leonardi *et al.* 2018, Macedo *et al.* 2019).

While the LH response was lower than that observed during a preovulatory surge elicited by the positive feedback effect of oestrogen (40–50 ng/mL; NIH-LH-B9 standard; Dieleman *et al.* 1983, 1986), these data demonstrate that by 4 weeks postpartum there are releasable stores of GnRH in the hypothalamus of anovulatory dairy cattle. This is a somewhat novel finding to our knowledge. Furthermore, these releasable stores are capable of stimulating sufficient LH to initiate events that lead to ovulation through the positive feedback system involving endogenous oestrogen. This is synonymous to the case in the ewe, in which Kp treatment activates the hypothalamic-pituitary axis and an LH surge and ovulation ensue beyond the time of treatment. It is possible that Kp acted directly on the pituitary to stimulate the observed increase in LH, although studies in sheep suggest this to be unlikely. While ovine gonadotropes express the Kp receptor, Kp did not have a direct effect on the secretion of LH (Smith *et al.* 2008), and Kp had only a minor effect on LH and FSH secretion from bovine pituitary cells in culture (Ezzat *et al.* 2010). The most likely site of action of the Kp agonist is at the level of GnRH terminals in the median eminence, which is outside the blood–brain barrier (Ezzat *et al.* 2015). The magnitude of the response to the chosen dose of Kp agonist was similar to that obtained in heifers with an intravenous injection of 1 mg of human Kp (Kadokawa *et al.* 2008) but lower than that obtained with a dose of 250 μg GnRH (McDougall *et al.* 1995*b*).

Gonadotrophin secretion in response to the second Kp injection in the Kp-Kp group was not observed in the current study, possibly due to the depletion of releasable stores of either GnRH from the hypothalamus or LH and FSH from the pituitary. An alternate possibility is the downregulation of either the Kp receptor in the GnRH neurons or the GnRH receptors in the pituitary. Leonardi *et al.* (2020) demonstrated that multiple doses of native Kp administered over a period of 2 h does maintain LH responsiveness to Kp. Differences in form and dosage of the Kp used likely account for the state of refractiveness that cows of the current study demonstrated at 12 h after the first Kp treatment.

A particularly interesting observation was that all but one of the Kp-induced ovulations was followed with a normal-length cycle. On the other hand, McDougall *et al.* (1995*b*) reported that short cycles and failure of cows to maintain ovulatory cycles occurred after GnRH agonist treatment in postpartum anovulatory cows. This could be associated with study of animal differences, or the effect could be related to the actions of Kp. Premature release of PGF2α from the uterus, in association with greater numbers of oxytocin receptors in the endometrium, is responsible for short cycles (Rhodes *et al.* 2003) like those observed following spontaneous first ovulations in the current study. It is unknown to us how the Kp treatment would interact with this mechanism to promote normal-length cycles characteristic of the cows having been progesterone primed before first postpartum ovulation.

Increased LH pulse frequency is an important driver of first ovulation in postpartum cattle (Lamming *et al.* 1981, Beam & Butler 1997). A lack of increased LH pulse frequency with bolus administration of Kp is one possible explanation for there being no apparent advantage to those cows receiving Kp without the presence of mature LH-responsive dominant follicle present in the ovaries. More intensive sampling would be required to determine whether pulsatile LH secretion is induced by bolus Kp treatment, but this was not undertaken in the present study in order to be minimally invasive. Continuous infusion of Kp restores pulsatile LH secretion in ewes treated with a neurokinin B antagonist (Clarke *et al.* 2018) as well as increasing the pulsatile secretion of GnRH and LH in anoestrous ewes (Caraty *et al.* 2013). Furthermore, a 24-h continuous infusion of a neurokinin B (a neuropeptide that stimulates Kp neurons) agonist increases LH pulse frequency in early postpartum dairy cows (Nakamura *et al.* 2017). The latter approach was effective in advancing the onset of ovulatory cycles among the entire treated population, in contrast to the results of the current study. Whether this can be achieved with a non-continuous, bolus-type delivery remains unknown but is an important consideration for it to be sufficiently practical for implementation on dairy farms.

In conclusion, we show that the Kp agonist used in this study stimulated an elevation in LH and FSH lasting several hours in postpartum anovulatory cows, but the ovulatory response depended upon the stage of the ovarian follicle wave. Kp-induced ovulation commonly resulted in oestrous cycles of normal length, which is atypical for cows having their first postpartum oestrous cycle.

## Declaration of interest

The authors declare that there is no conflict of interest that could be perceived as prejudicing the impartiality of the research reported.

## Funding

This work was funded through a partnership (DRCX1302) between the New Zealand Ministry of Business, Innovation and Employment and New Zealand dairy farmers through DairyNZ Inc.

## Author contribution statement

I C and C B conceived the study and wrote the paper. C B conducted the study with technical support. R M provided the Kp agonist. J R and R M provided critical input into the paper. J R served as programme manager.
